# *Arctium lappa* ameliorates endothelial dysfunction in rats fed with high fat/cholesterol diets

**DOI:** 10.1186/1472-6882-12-116

**Published:** 2012-08-06

**Authors:** Yun Jung Lee, Deok Ho Choi, Guk Hyun Cho, Jin Sook Kim, Dae Gill Kang, Ho Sub Lee

**Affiliations:** 1 College of Oriental Medicine and Professional Graduate School of Oriental Medicine, Wonkwang University, Shinyong-dong, Iksan, Jeonbuk 570-749, Republic of Korea; 2 Hanbang Body-fluid Research Center, Wonkwang University, Shinyong-dong, Iksan, Jeonbuk 570-749, Republic of Korea; 3 Korea Institute of Oriental Medicine, Jeonmin-dong, Yusung-gu, Daejeon 305-811, Republic of Korea

**Keywords:** Arctium lappa, Hyperlipidemia, Hypertension, Vasorelaxation, Inflammation

## Abstract

**Background:**

*Arctium lappa* L. (Asteraceae), burdock, is a medicinal plant that is popularly used for treating hypertension, gout, hepatitis, and other inflammatory disorders. This study was performed to test the effect of ethanol extract of *Arctium lappa* L. (EAL) seeds on vascular reactivity and inflammatory factors in rats fed a high fat/cholesterol diet (HFCD).

**Method:**

EAL-I (100 mg·kg^−1^/day), EAL-II (200 mg·kg^−1^/day), and fluvastatin (3 mg·kg^−1^/day) groups initially received HFCD alone for 8 weeks, with EAL supplementation provided during the final 6 weeks.

**Results:**

Treatment with low or high doses of EAL markedly attenuated plasma levels of triglycerides and augmented plasma levels of high-density lipoprotein (HDL) in HFCD-fed rats. Chronic treatment with EAL markedly reduced impairments of acetylcholine (ACh)-induced relaxation of aortic rings. Furthermore, chronic treatment with EAL significantly lowered systolic blood pressure (SBP) and maintained smooth and flexible intimal endothelial layers in HFCD-fed rats. Chronic treatment with EAL suppressed upregulation of intercellular adhesion molecule (ICAM)-1, vascular cell adhesion molecule (VCAM)-1, and E-selectin in the aorta. Chronic treatment with EAL also suppressed increases in matrix metalloproteinase (MMP)-2 expression. These results suggested that EAL can inhibit HFCD-induced vascular inflammation in the rat model.

**Conclusion:**

The present study provides evidence that EAL ameliorates HFCD-induced vascular dysfunction through protection of vascular relaxation and suppression of vascular inflammation.

## Background

Vascular tone is an important factor in the regulation of arterial blood pressure. Changes in vascular smooth muscle tone and the internal diameter of vessels can profoundly alter tissue perfusion and can impair the ability of arteries to respond to vasodilators and vasoconstrictors [1,2]. Endothelium-dependent vasorelaxation is mediated by nitric oxide (NO), which acts through soluble guanylyl cyclase and cGMP. This phenotypic change is associated with NO bioavailability, and reduction in NO biosynthesis and inactivation of NO by superoxide lead to hypertension [3]. Hypertension, an impaired vascular response, has been identified as an independent risk factor for the development of endothelial dysfunction and inflammation [4]. Mouse or rat models fed with high fat/cholesterol diet (HFCD) have been used to study these vascular phenotypes [5,6]. Impaired relaxation of the aorta induced by acetylcholine in obese rats is a consequence of endothelial dysfunction [7]. HFCD causes an unbalanced lipoprotein metabolism and leads to hyperlipidemia, characterized by high levels of serum triglyceride and total cholesterol [8]. Many epidemiological, clinical, and experimental studies have indicated that reducing elevated serum low-density lipoprotein (LDL) levels is an effective way to prevent atherosclerosis and cardiovascular diseases [9].

An early phase of atherosclerosis involves recruitment of inflammatory cells from the circulation and their transendothelial migration [10]. This process is predominantly mediated by cellular adhesion molecules, which are expressed on the vascular endothelium and on circulating leukocytes in response to several inflammatory stimuli. Selectins (P, E, and L) and their ligands are involved in the rolling and tethering of leukocytes on the vascular wall. Intracellular adhesion molecule-1 (ICAM-1) and vascular cell adhesion molecule (VCAM-1) induce firm adhesion of inflammatory cells at the vascular surface [11].

*Arctium lappa* L. (Asteraceae), burdock, is a medicinal plant that is popularly used for treating hypertension, gout, hepatitis and other inflammatory disorders, and it is also used as a diuretic and antipyretic tea. The roots are widely used as a food, whereas the seeds are used in traditional Korean medicine as a diuretic, anti-inflammatory, or detoxifying agent [12]. The root contains at least 5 powerful flavonoid-type antioxidants (i.e. caffeoylquinic acid derivatives) and several polyphenols [13]. The seed contains platelet activating factor (PAF) inhibitors that may reduce symptoms of PAF-related diseases such as arthritis and asthma [14]. Burdock seed also contains polyacetylenes that have antibacterial, antifungal, and anti-HIV activity, and tannins [15]. However, although the seeds of *A. lappa* have been used as an alternative medicine in Korea for the treatment of inflammatory disorders, little information is available concerning the pharmacological basis of their activity on vascular function. Therefore, we investigated the effects of an ethanol extract of *A. lappa* (EAL) on vascular dysfunction in HFCD-fed rats.

## Methods

### Preparation of EAL

The seeds of *A. lappa* were purchased from the Herbal Medicine Cooperative Association, Jeonbuk Province, Korea. The herbarium voucher specimen (No. HBH071) was deposited in the herbarium of the Professional Graduate School of Oriental Medicine (Wonkwang University, South Korea). Dried seeds of *A. lappa* (600 g) were extracted with 2,000 mL of 95% ethanol at 24°C for 1 week. The extract was filtered through Whatman No. 3 filter paper and concentrated using a rotary evaporator (N-1000 S, EYELA, Japan). The resulting extract (4.99 g) was lyophilized using a freeze-drier and retained until required.

### Experimental animals

All animal procedures were in strict accordance with the National Institutes of Health Guide for the Care and Use of Laboratory Animals and were approved by the Institutional Animal Care and Utilization Committee for Medical Science of Wonkwang University. Forty male Sprague–Dawley (SD) rats at age 8 weeks and ranging from 240–290 grams were obtained from Samtako (Osan, Korea) and were housed in metabolic cages with an automatically controlled temperature (22 ± 2°C), relative humidity (50–60%), and light (12 h light/dark cycle). Throughout the experiments, all animals had unrestricted access to water. After 2 weeks acclimatization, animals were randomly divided into 5 groups (n = 8 per group): Control (regular diet); HFCD; Fluvastatin (HFCD + 3 mg·kg^−1^/day of fluvastatin); EAL-I (HFCD + 100 mg·kg^−1^/day of EAL); and EAL-II (HFCD + 200 mg·kg^−1^/day of EAL). The control group was given a standard laboratory chow diet (regular diet, RD) for 14 weeks (D10012M, Research Diets, New Brunswick, NJ). The HFCD group was fed a diet containing 7.5% cocoa butter and 1.25% cholesterol mix (D12451, Research Diets) for 14 weeks. The fluvastatin, EAL-I, and EAL-II groups initially received HFCD alone for 8 weeks, with supplementation with EAL or fluvastatin occurring during the final 6 weeks.

### Measurement of blood pressure

Systolic blood pressure (SBP) was determined by a tail-cuff plethysmography method and recorded with an automatic sphygmotonograph (Muromachi Kikai, Tokyo, Japan). At least 8 determinations were made in every session and the mean of the lowest 5 values within 5 mmHg was recorded as the SBP.

### Biochemical analysis

Plasma glucose, HDL, LDL, triglyceride, blood urea nitrogen (BUN), creatinine, total bilirubin, albumin, and glutamic oxaloacetic transaminase (GOT) levels were enzymatically measured using commercially available kits (Arkray Factory Inc., Kyoto, Japan).

### Recording of isometric vascular tone

The method of measuring vascular tone was performed as described previously by Kang *et al.*[16]. At the end of the experiment, rats were sacrificed by decapitation. The thoracic or carotid aorta was rapidly and carefully dissected and placed into ice-cold Kreb’s solution (118 mM NaCl, 4.7 mM KCl, 1.1 mM MgSO_4_, 1.2 mM KH_2_PO_4_, 1.5 mM CaCl_2_, 25 mM NaHCO_3_, and 10 mM glucose; pH 7.4). The aortas were separated from connective tissue and fat and sectioned into rings with a width of approximately 3 mm. All dissection was carried out with extreme care to protect the endothelium from inadvertent damage. The aortic rings were suspended in a tissue bath containing Kreb’s solution at 37°C by means of 2 L-shaped stainless-steel wires inserted into the lumen. A gas mixture of 95% O_2_ and 5% CO_2_ was continuously bubbled through the bath. The baseline load placed on the aortic rings was 1.0 *g*. Changes in isometric tension were recorded using a Grass FT 03 force displacement transducer connected to a Model 7E polygraph recording system (Grass Technologies, Quincy, MA). Aortic relaxation by cumulative addition of acetylcholine was performed in the presence of endothelium.

### Protein preparation and Western blot analysis

Thoracic aortas were homogenized in a buffer consisting of 250 mM sucrose, 1 mM EDTA, 0.1 mM phenylmethylsulfonyl fluoride, and 20 mM potassium phosphate buffer (pH 7.6). Large tissue debris and nuclear fragments were removed by successive low speed spins (3,500 rpm, 5 min; 8000 rpm, 10 min; 4°C). The recovered protein (40 μg) was separated by 10% sodium dodecyl sulfate-polyacrylamide gel electrophoresis (SDS-PAGE) and transferred electrophoretically to nitrocellulose membranes using a Mini-Protean II apparatus (Bio-Rad, Hercules, CA). A SDS-PAGE protein standard was used to check transfer efficiency and as a molecular weight marker. Membranes were blocked with 5% non-fat milk powder in 0.05% Tween 20-phosphate buffered saline (PBST) for 1 h prior to overnight incubation at 4°C in the presence of primary antibodies to Akt1/2/3 or β-actin (Santa Cruz Biotechnology, Santa Cruz, CA) at a final dilution of 1:1000. The blot was washed several times with PBST and incubated with the appropriate horseradish peroxidase-conjugated secondary antibody for 1 h. After the membrane was washed several times with PBST, the bound secondary antibody was detected by enhanced chemiluminescence (Amersham, Buckinghamshire, UK). Protein expression levels were determined by analyzing the signals captured on the nitrocellulose membrane using a Chemi-Doc image analyzer (Bio-Rad).

### Quantitative histopathology

Aortas isolated from all groups were fixed in 10% (v/v) formalin in 50 mM potassium phosphate buffer (pH 7.0) for 48 h at 4°C. The tissues were subsequently embedded in paraffin and cross-sections (6 μm) of the aortic arch in each group were stained with hematoxylin and eosin (H&E) [17]. For quantitative histopathologic comparisons, the mean of 10 sections was taken and the intima–to-media ratio was determined by Axiovision 4 Imaging/Archiving Software (Axiovision 4, Carl Zeiss, Germany). The derangement of intima was indicated by arrow.

### Immunohistochemistry

Sections were stained after incubation with 5% normal goat serum for 10 min at room temperature to reduce non-specific background staining. ICAM-1 and VCAM-1 (Oncogene, Cambridge, MA) antibodies were added as a 1:500 dilution and specimens were incubated in humidified chambers overnight at 4°C. All slides were then sequentially incubated with biotinylated secondary antibody and horseradish peroxidase-conjugated streptavidin, both for 10 min at room temperature. Peroxidase activity was visualized by the 3-amino-9-ethylcarbazole substrate-chromogen system (Zymed, San Francisco, CA), which resulted in brownish-red staining. Representative sections were photographed by Axiovision 4 Imaging/Archiving Software.

### Statistical analyses

Values are shown as mean ± SE. Statistical analyses were performed using analysis of variance followed by the Student’s *t-test* for unpaired data and one-way ANOVA followed by Bonferroni’s multiple-comparison test. Differences with a p value of <0.05 were considered statistically significant.

## Results and discussion

The present study constitutes the first report of evidence that EAL ameliorates the development of atherosclerosis, possibly by decreasing vascular endothelial inflammation in HFCD rats. Two doses of EAL (100 and 200 mg·kg^−1^/day) and fluvastatin (3 mg·kg^−1^/day), which is one of the 3-hydroxy-3-methylglutaryl-CoA (HMG-CoA) reductase inhibitors (statins), were tested. Groups initially received HFCD alone for 8 weeks, with EAL or statin administration occurring during the final 6 weeks. During the 14 weeks of the HFCD regimen, cumulative food intake among the 5 groups was not significantly different (p>0.05) (data not shown). No mortality was observed and EAL was found to be safe at the given doses. The HFCD-fed SD rats developed a severe metabolic syndrome consistent with hypertension and hypercholesterolemia [18,19]. The potential toxicity of EAL was determined based on BUN, creatinine, albumin, GOT, and GPT levels in the plasma. Table 1 shows that renal function with regard to BUN and creatinine was unchanged in the HFCD rats at 14 weeks. In addition, the liver function parameters albumin, GOT, and GPT, did not show any sign of toxicity.

**Table 1:** Effects of EAL on renal and liver function

	**BUN (mg/dL)**	**Cre (mg/dL)**	**Alb (g/dL)**	**GOT (IU/L)**	**T-Bil (mg/dL)**
HFCD	13.0 ± 0.71	0.6 ± 0.05	2.9 ± 0.05	192.8 ± 8.84	0.23 ± 0.02
Fluva	13.3 ± 0.48	0.6 ± 0.06	3.2 ± 0.06	197.3 ± 21.25	0.27 ± 0.02
EAL I	13.3 ± 0.63	0.6 ± 0.03	2.9 ± 0.10	182.6 ± 20.54	0.20 ± 0.00
EAL II	14.3 ± 0.48	0.5 ± 0.09	2.6 ± 0.08	181.0 ± 34.64	0.22 ± 0.02

Blood urea nitrogen (BUN), creatinine, albumin, glutamic oxaloacetic transaminase (GOT), and total bilirubin levels were enzymatically measured. Values are expressed as mean ± S.E. of 3 individual experiments.

### EAL effects on endothelial dysfunction: vascular relaxation

**Figure 1:**
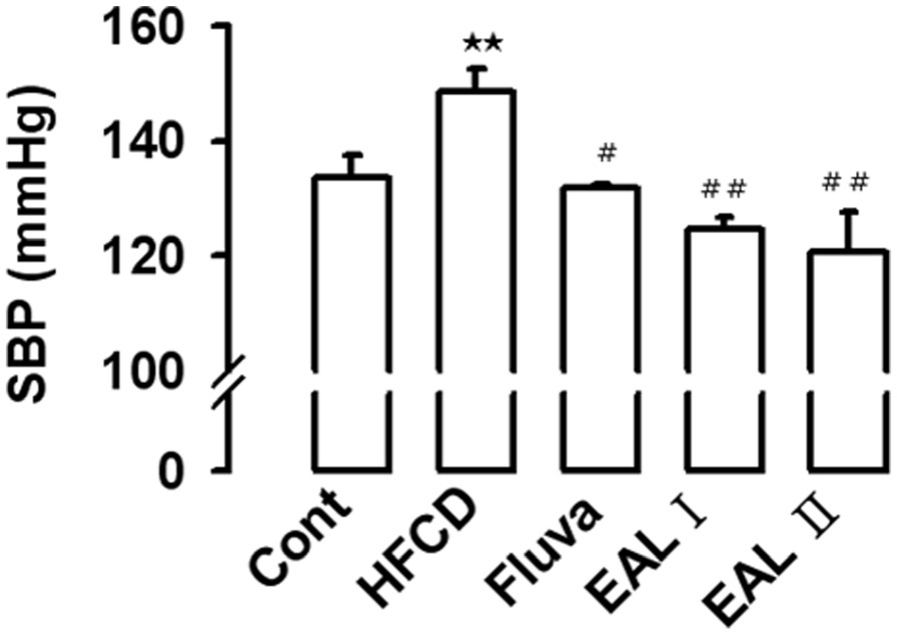
**Effect of EAL on SBP of HFCD-fed rats.** Values are expressed as mean ± S.E. (n = 8). Cont, Control; HFCD, High fat/cholesterol diet; Fluva, Fluvastatin; EAL I, EAL 100 mg·kg^−1^/day; EAL II, EAL 200 mg·kg^−1^/day. **p<0.01 vs. Control; #p<0.05, ##p<0.01 vs. HFCD.

**Figure 2:**
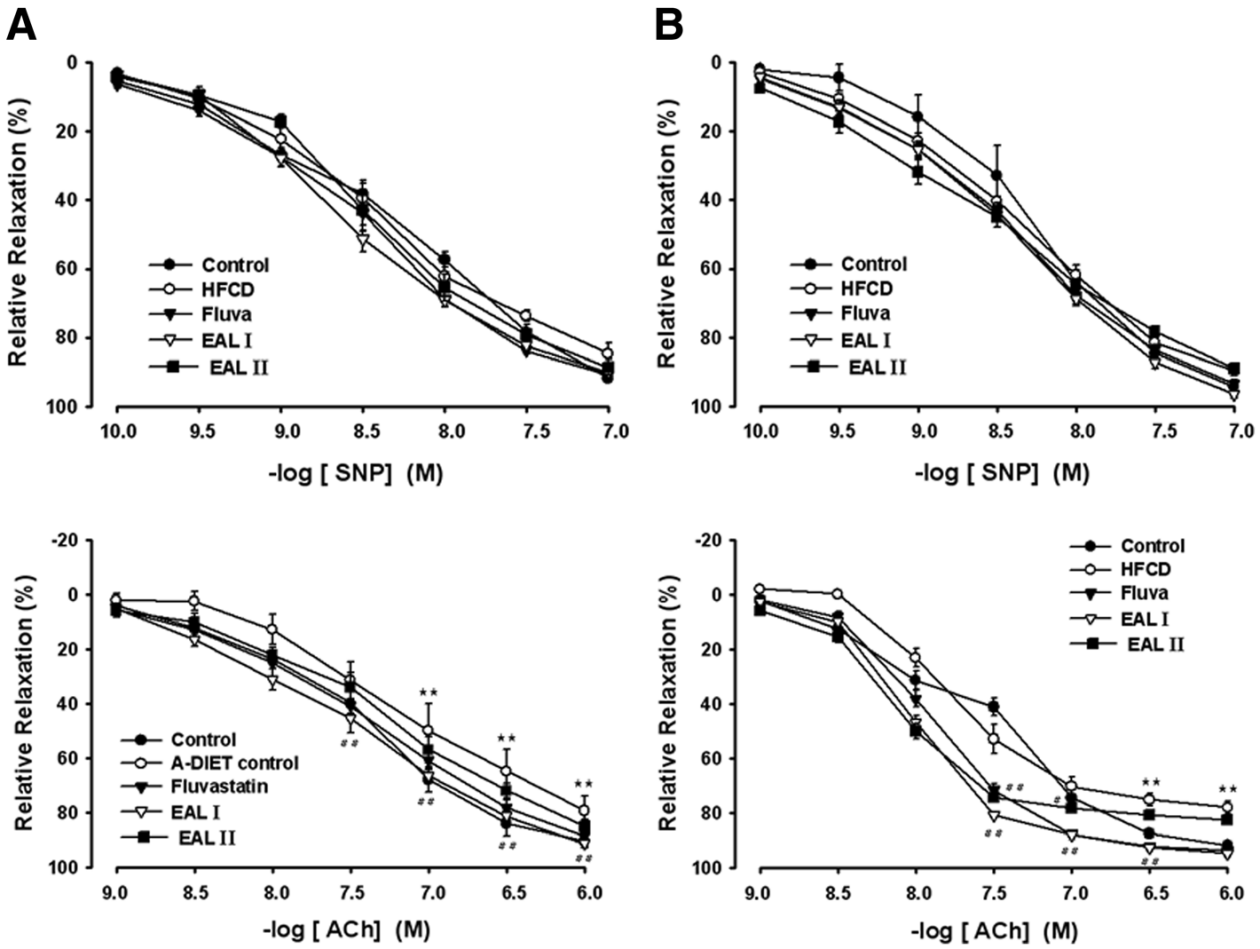
**Effects of EAL on Acetylcholine or SNP-induced relaxation of carotid (A) and thoracic (B) aorta in HFCD-fed rats.** Values are expressed as mean ± S.E. (n = 8); **p<0.01 vs. Control; #p<0.05, ##p<0.01 vs. HFCD.

The endothelium can sense changes or abnormalities in blood flow and pressures, and the vascular endothelium that exists between circulating blood and vascular smooth muscle plays an important role in modulation of vascular tone [20]. In our results, blood pressure was determined using the tail-cuff technique (Figure 1). The mean SBP in rats with 14 weeks of HFCD was significantly increased as compared with RD-fed rats, however, 100 and 200 mg·kg^−1^/day EAL and fluvastatin treatment all significantly decreased this trend. HFCD also led to endothelial dysfunction, as evidenced by a decreased response to ACh-induced vascular relaxation. Figure 2 shows the vasorelaxant responses to acetylcholine in carotid and thoracic aortas of HFCD-fed rats. Significant impairment of vasorelaxation was evident in both the carotid and thoracic aorta in the HFCD-fed rats (p<0.01 vs. RD-fed rats). Doses of 100 and 200 mg·kg^−1^/day EAL and fluvastatin treatment all resulted in significant recovery of the vasorelaxant response to acetylcholine (p<0.01) (Figure 2A). On the other hand, the vasorelaxant response to sodium nitroprusside (SNP), a NO donor, was unchanged in both the carotid and thoracic aorta, and EAL and fluvastatin did not affect this response (Figure 2B). These findings suggested that the hypotensive effect of EAL is mediated by ACh and further via an endothelium-dependent NO/cGMP pathway. In fact, other studies have also reported defective acetylcholine response without a corresponding change in SNP response in aortas of obese rats fed a high fat diet, and impaired relaxation of the aorta induced by acetylcholine but not SNP has been seen in obese Zucker rats as a consequence of endothelial dysfunction [21,22]. Since the endothelium-dependent ability of NO to maintain vascular tone is deficient in endothelial dysfunction, endothelium-dependent vasorelaxation is impaired in both hypercholesterolemia and atherosclerosis [23-25]. Recently, it has also been shown that fluvastatin ameliorates endothelial dysfunction and hypercontractility of vascular myocytes in obese Zucker rats [7]. Fluvastatin also consistently reduced SBP and diet-induced defects in ACh-mediated vasorelaxation in our animal models. Other recent studies have demonstrated that high-fat diets associated with insulin resistance and endothelial dysfunction precede the development of hypertension [26,27]. In the current study, plasma blood glucose levels were not statistically different in HFCD-fed rats with chronic treatment of EAL (Table 2); this result indicated that HFCD-induced hypertension was independent of insulin resistance, and the finding again suggested the protective role of EAL on diet-induced hypertension and vasoconstriction.

**Table 2:** Effect of EAL on plasma triglyceride, LDL, HDL, and glucose levels in HFCD rats

	**Triglyceride (mg/dL)**	**LDL (mg/dL)**	**HDL (mg/dL)**	**Glucose (mg/mL)**
HFCD	67.2 ± 4.31	120.8 ± 3.88	20.8 ± 1.36	97.8 ± 1.85
Fluva	34.6 ± 3.12^##^	107.4 ± 7.20	25 ± 1.84	105.6 ± 1.78
EAL I	33.8 ± 1.71^##^	113.4 ± 4.08	25 ± 1.30^#^	98.2 ± 2.15
EAL II	27.2 ± 1.98^##^	119 ± 6.20	42.6 ± 2.80^##^	92.2 ± 1.46

Values are expressed as mean ± S.E. of 3 individual experiments. ^#^p<0.01, ^##^p<0.001 vs. HFCD.

### EAL and lipid metabolism

Blood samples were analyzed biochemically to evaluate changes in lipid metabolism in the HFCD-fed rats (Table 2 ). Treatment with EAL (100 and 200 mg·kg^−1^/day) significantly decreased triglyceride levels compared with HFCD-fed rats (p<0.01). Long-term feeding with HFCD had no effect on plasma LDL levels; however, rats treated with EAL had significantly elevated HDL levels. Fluvastatin, as a positive control, also decreased triglyceride levels and increased HDL levels without LDL alteration. Chronic treatment with EAL significantly decreased HFCD-induced elevations in triglyceride levels and increased HDL-cholesterol levels. Elevated LDL-cholesterol levels impair endothelial function, and LDL-cholesterol deposited in blood vessel wall forms part of the atherosclerotic plaque [28,29]. As noted, there was no change of LDL cholesterol levels in the EAL treatment groups. This discrepancy suggested a direct correlation between circulating levels of HDL cholesterol and a reduction in the potential for atherosclerosis. We also could not rule out the possible role of cholesterol ester transfer protein (CETP) in this effect. Dalcetrapib, a CETP inhibitor, has been found to increase HDL levels (19–37%) and modestly decrease LDL levels (~6%) [30], while the CETP inhibitor anacetrapib resulted in a significant increase in both HDL (~130%) and LDL (40%) levels [31,32]. The significant distinction between the various CETP inhibitors that cause different regulation of cholesterol levels led us to speculate that EAL might be involved in CETP regulation, resulting in the increase of HDL. These findings, at least in part, indicate that EAL also protects against initiation and development of atherosclerosis by improving lipid metabolism.

### EAL and vascular morphology

**Figure 3:**
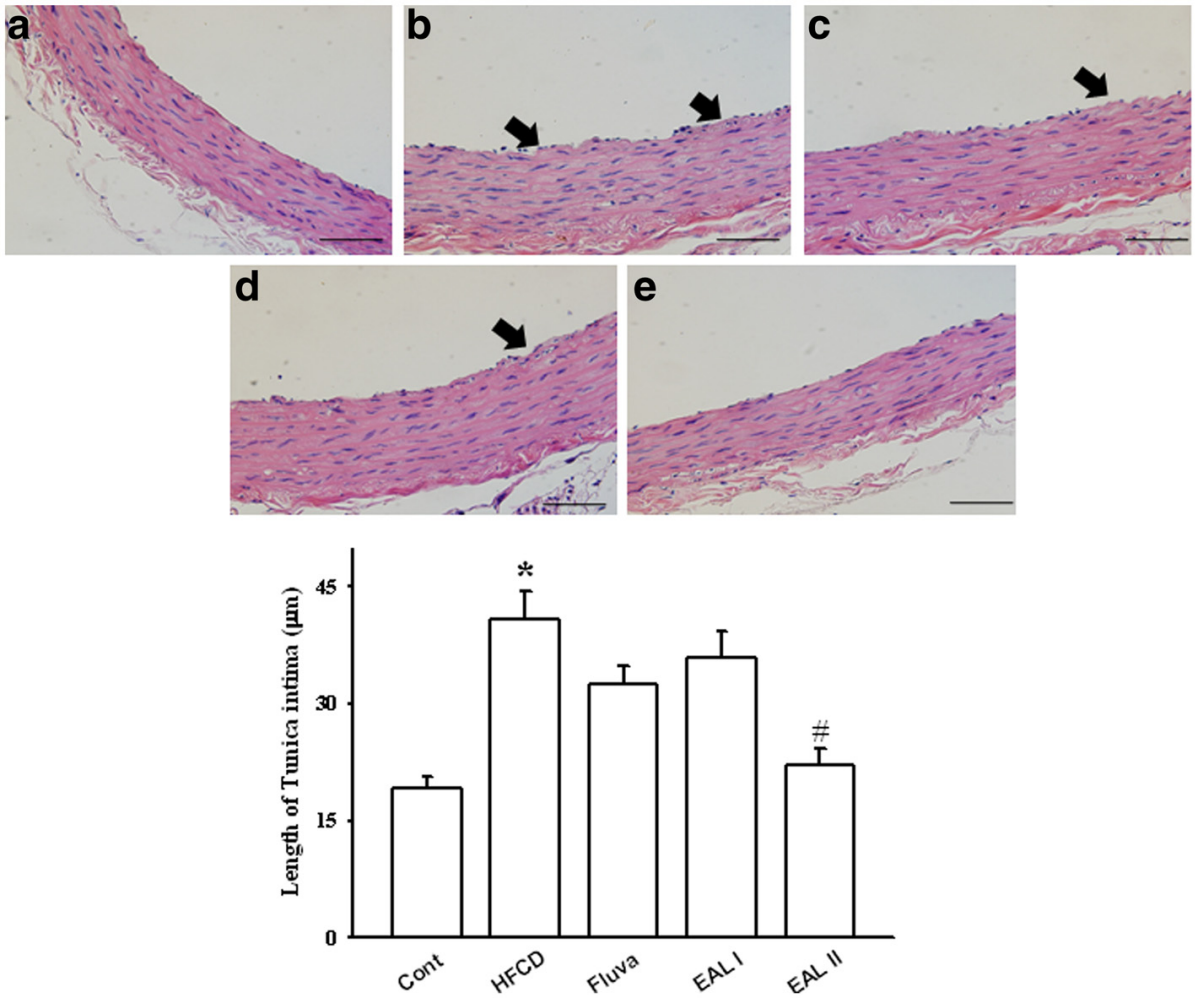
**Representative photomicrographs of H&E-stained sections of thoracic aorta of HFCD-fed rats.** Aortas are from: control (**a**), HFCD (**b**), fluvastatin (**c**), EAL I (**d**), and EAL II (**e**) groups. The scale bar represents 800 μm. The lower panels show intima thickness. Values are expressed as mean ± S.E. (n = 8); *p<0.05 vs. control; ^#^p<0.05 vs. HFCD alone. Arrows indicate deranged intima.

It is implied that endothelial dysfunction will include not only reduced vasodilation but also inflammation and atherosclerotic lesions [33,34]. Blocking of inflammatory mediators can decrease the size of the atherosclerotic lesion. We hypothesized that the vasorelaxant effect of EAL would contribute anti-inflammatory and anti-atherosclerotic effects in rats with atherogenic diets. Microscopic examination of arterial specimens with H&E staining revealed that supplementation with EAL significantly reduced thickening of the tunica intima layer and decreased the size of atherosclerotic lesions found in HFCD-fed rats. Chronic treatment with fluvastatin and EAL I and II maintained the smoothness of the intimal endothelial layers (Figure 3). Previous histological analysis has demonstrated that rougher intimal endothelial layers in aortic sections of HFCD-fed rats were associated with a trend towards a thickened medial layer [35]. Thus, HFCD can induce thickening of the aortic intima-media that is compatible with the processes of atherosclerosis and intimal derangement, and our experiments showed that these morphological changes could be prevented by EAL treatment.

### EAL and vascular inflammatory markers

**Figure 4:**
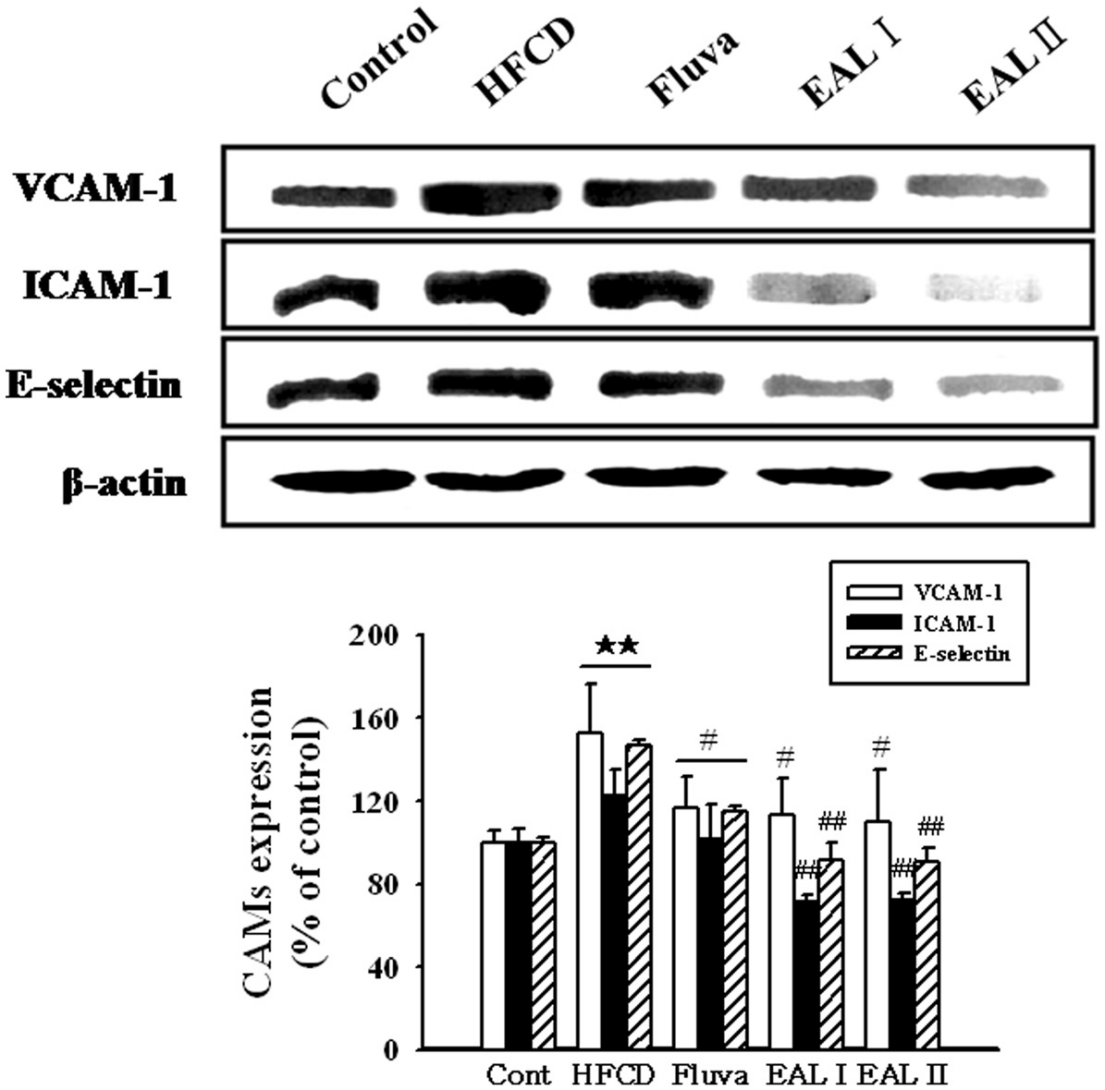
**Effects of EAL on expression of adhesion molecules in the thoracic aorta.** Representative western blots (upper panel) of VCAM-1, ICAM-1, and E-selectin expression in aortic tissues, and quantification (bottom panel) are shown. Each photograph is representative of the results from 5 independent experiments. The scale bar represents 15 μm (Magnification, ×400). Values are expressed as a percentage of the density of blot (mean ± S.E.); **p<0.01 vs. Control; ^##^p<0.01 vs. HFCD alone.

**Figure 5:**
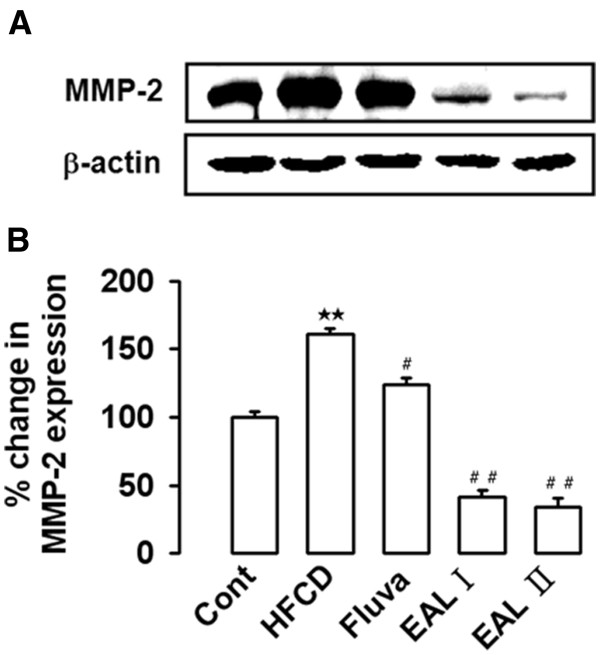
**Effect of EAL on MMP-2 expression in the aorta of HFCD-fed rats.** Representative western blot analysis and quantification are shown. Lower panel indicated densitometric quantification normalized by actin. Each photograph is representative of the results from 5 independent experiments. **p<0.01 vs. control; #p<0.05, ##p<0.01 vs. HFCD.

**Figure 6:**
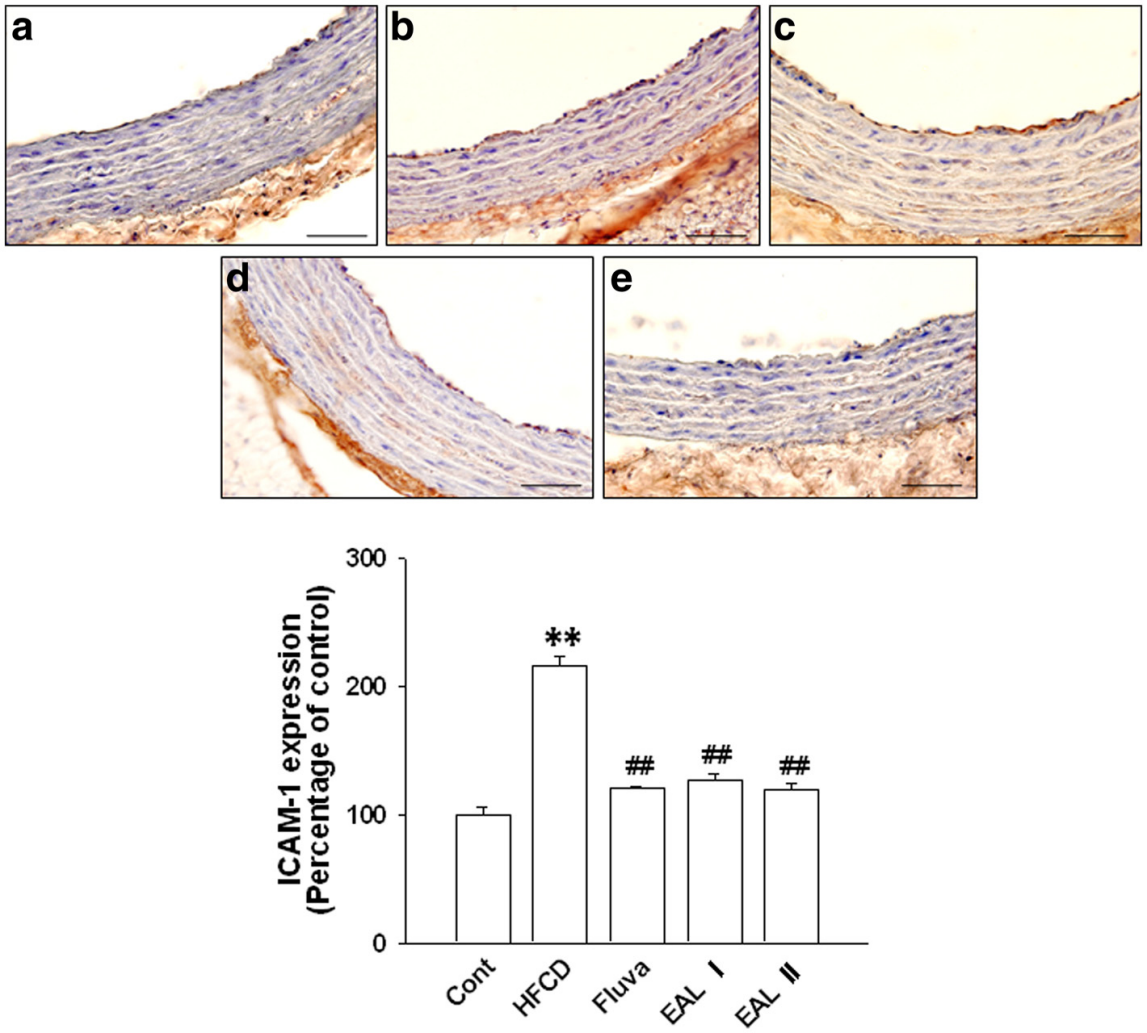
**ICAM-1 immunohistochemistry in the thoracic aorta.** Control (**a**); HFCD group (**b**); fluvastatin-treated HFCD group (**c**); EAL I-treated HFCD group (**d**); EAL II-treated HFCD group (**e**). The lower panel shows quantitative analysis of the ICAM-1-positive area. The average score of 5–10 randomly selected sites per section of aorta was calculated. Data are expressed as mean ± S.E.; **p<0.01 vs. control; #p<0.05, ##p<0.01 vs. HFCD. The scale bar represents 15 μm (Magnification, ×400).

**Figure 7:**
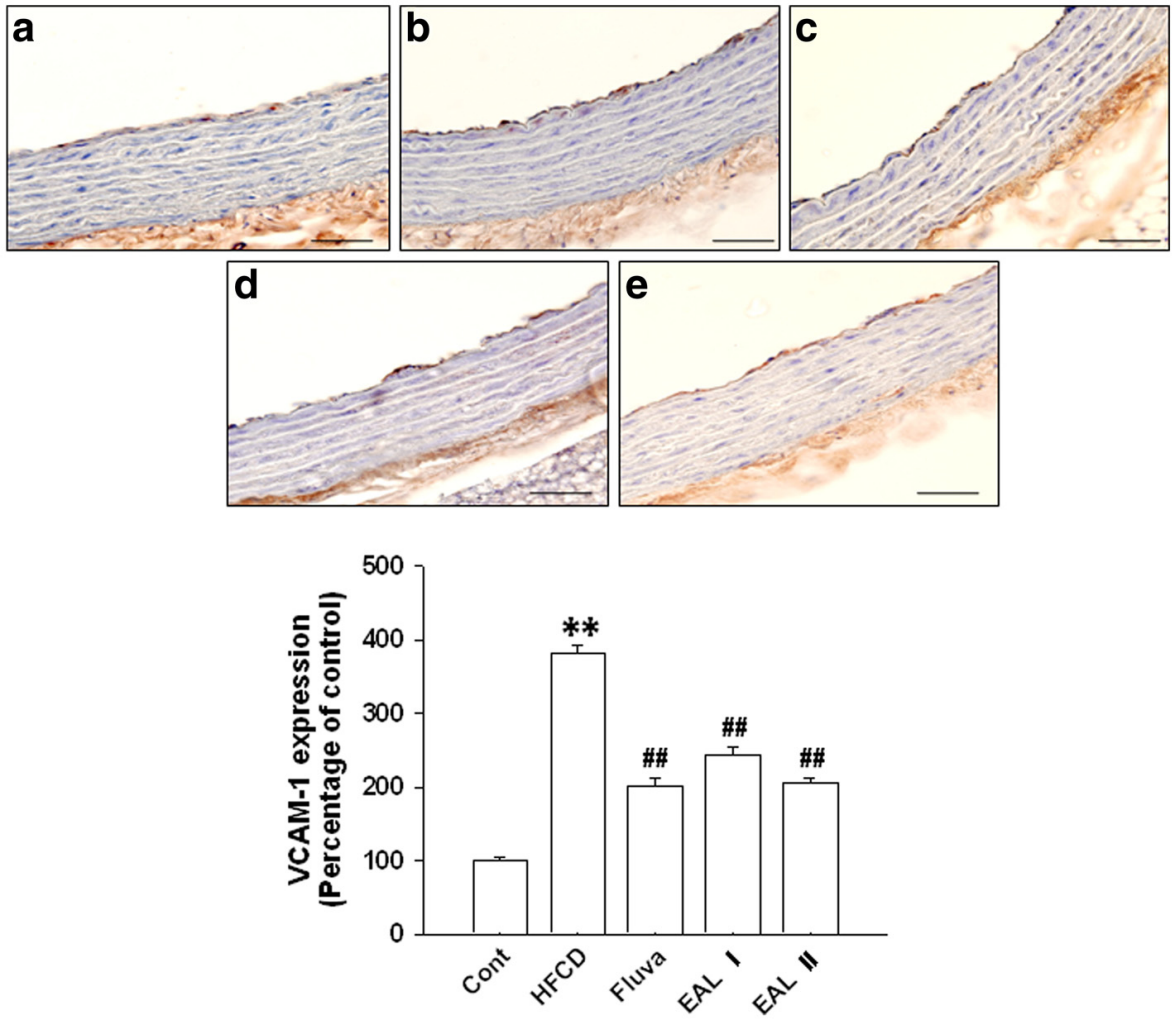
**VCAM-1 immunohistochemistry in the thoracic aorta.** Control (**a**); HFCD group (**b**); fluvastatin-treated HFCD group (**c**); EAL I-treated HFCD group (**d**); EAL II-treated HFCD group (**e**). The lower panel shows the quantitative analysis of the VCAM-1-positive area. The average score of 5–10 randomly selected sites per section of aorta was calculated. Data are expressed as mean ± S.E.; **p<0.01 vs. Control; #p<0.05, ##p<0.01 vs. HFCD. The scale bar represents 15 μm (Magnification, ×400).

Activation of the endothelium at sites of inflammation allows numerous leukocytes to adhere to the vascular endothelium, transmigrate of the endothelium, and aggravate endothelial dysfunction and tissue injury [36,37]. Leukocyte infiltration at the sites of inflammation is regulated in part by specific endothelial-leukocyte adhesion molecules including VCAM-1, ICAM-1, and E-selectin [38]. Meanwhile, the activated macrophage is considered the most important MMP producer in atherosclerotic plaques [39,40]. To examine the effect of EAL on vascular inflammation, adhesion molecules including VCAM-1, ICAM-1, and E-selectin were measured by western blot analysis using the tissues of the thoracic aorta. The HFCD rats had significantly increased levels of aortic expression of VCAM-1, ICAM-1, and E-selectin. However, in comparison, the expression levels of these proteins were significantly reduced in a dose-dependent manner in the EAL I and EAL II treatment groups (Figure 4). MMP-2 expression was also increased in the HFCD rats, and EAL was found to decrease HFCD-induced MMP-2 expression (Figure 5). MMP-2 (gelatinase) is known to stimulate subintimal smooth muscle cell migration and macrophage aggregation [41]. The reduction of the atherosclerotic lesions in our study might be attributed to prevention of smooth muscle cell and monocyte migration into the intima by inhibition of MMP-2 expression; thus, the decrease of MMP-2 attributed to EAL may be one of the therapeutic benefits of its anti-atherosclerotic properties. The levels of expression of endothelial VCAM-1 and ICAM-1 in the thoracic aorta were determined by immunohistochemical analysis, and the HFCD group had increased aortic endothelial ICAM-1 expression compared with the groups treated with EAL, which showed comparative decreases in these levels in all areas of the aorta (Figure 6). VCAM-1 was also detected in tunica intima of the thoracic aorta segment in HFCD rats. EAL treatment blocked these increases in VCAM-1 expression levels (Figure 7). These findings suggest a potentially important role for EAL in anti-inflammatory and anti-atherosclerotic activity when hyperlipidemia and/or hypertension are present.

## Conclusions

Though *A. lappa* has been a popular medicine worldwide, the pharmacologic mechanisms of the seeds are unknown. Treatment of HFCD-fed rats with EAL reduced hypertension by protection of the endothelium-dependent vasorelaxation response in HFCD rats. EAL also improved HDL cholesterol and triglyceride levels and reduced expression of vascular inflammation markers. As a result, EAL prevented HFCD-induced atherosclerosis. To our knowledge, this study is first to demonstrate apparent anti-hypertensive, hypolipidemic, and vascular anti-inflammatory effects of EAL in an animal model of atherosclerosis.

## Abbreviations

ACh: Acetylcholine; EAL: Ethanol extract of *Arctium lappa* L; HDL: High-density lipoprotein; HFCD: High fat/cholesterol diet; ICAM-1: Intercellular adhesion molecule-1; LDL: Low-density lipoprotein; MMP: Matrix metalloproteinase; SNP: Sodium nitroprusside; VCAM-1: Vascular cell adhesion molecule-1.

## Competing interests

The authors declare that they have no competing interests.

## Authors’ contributions

LYJ designed research; CDH and CGH conducted research and analyzed data; KDG wrote the paper; KJS and LHS had the primary responsibility for final content. All authors read and approved the final manuscript.

## Pre-publication history

The pre-publication history for this paper can be accessed here:

http://www.biomedcentral.com/1472-6882/12/116/prepub
